# Prediction of In-Hospital Cardiac Arrest Using Shallow and Deep Learning

**DOI:** 10.3390/diagnostics11071255

**Published:** 2021-07-13

**Authors:** Minsu Chae, Sangwook Han, Hyowook Gil, Namjun Cho, Hwamin Lee

**Affiliations:** 1 Department of Software Convergence, Soonchunhyang University, Asan 31538, Korea; cmspr90@gmail.com (M.C.); hsw89417@gmail.com (S.H.); 2 Department of Internal Medicine, Soonchunhyang University, Cheonan Hospital, Cheonan 31151, Korea; hwgil@sch.ac.kr (H.G.); chonj@schmc.ac.kr (N.C.); 3 Department of Computer Software Engineering, Soonchunhyang University, Asan 31538, Korea

**Keywords:** in-hospital cardiac arrest, machine learning, deep learning

## Abstract

Sudden cardiac arrest can leave serious brain damage or lead to death, so it is very important to predict before a cardiac arrest occurs. However, early warning score systems including the National Early Warning Score, are associated with low sensitivity and false positives. We applied shallow and deep learning to predict cardiac arrest to overcome these limitations. We evaluated the performance of the Synthetic Minority Oversampling Technique Ratio. We evaluated the performance using a Decision Tree, a Random Forest, Logistic Regression, Long Short-Term Memory model, Gated Recurrent Unit model, and LSTM–GRU hybrid models. Our proposed Logistic Regression demonstrated a higher positive predictive value and sensitivity than traditional early warning systems.

## 1. Introduction

During hospitalization, almost 3.7% of patients experience serious adverse events such as cardiopulmonary arrest, unplanned intensive care unit (ICU) admissions, and unexpected deaths [1]. The number of in-hospital cardiac arrests is increasing in the United States and the Republic of Korea [2,3]. However, several studies have reported that abnormal vital signs frequently precede these adverse events by several hours [4,5,6]. Many hospitals operate rapid response teams (RRTs), which use uses medical alert systems to respond quickly to such adverse events. There is evidence of decreased mortality and non-ICU cardiac arrest rates with the use of RRTs; however, the effects of RRTs on ICU transfer rates are equivocal [7]. Several risk scoring systems are used to identify patients at high risk of serious adverse events including unexpected inpatient death. More than 100 early warning systems (EWSs) are available to detect and manage clinical deterioration of patients, including the Modified Early Warning Score (MEWS), VitalPAC™ Early Warning Score (ViEWS), and the National Early Warning Score (NEWS) [8,9]. However, these systems have low sensitivities and specificities [10,11,12].

Vähätalo et al. studied the association between silent myocardial infarction (MI) and cardiac arrest [13]. They found that of 5869 cardiac arrest patients, 3122 (53.2%) had coronary artery disease without prior knowledge [13]; of these 3122 patients, 1322 (42.3%) had silent MI [13]. In addition, 67% of the patients had abnormal electrocardiography (ECG) findings before cardiac arrest [13]. Miyazaki et al. analyzed the records of 46 cardiac arrest patients aged 6 years or more and found that 21 (46%) had no history of arrhythmias [14]. In this study, we proposed a method to predict cardiac arrest in hospitalized patients by analyzing biosignals measured through patch-type sensors and lab code data based on shallow and deep learning.

There are few cases of applying shallow and deep learning to predict cardiac arrest. Kwon et al. [15] found that the sensitivities for predicting in-hospital cardiac arrest were 0.3%, 23%, and 19.3%, respectively, for MEWS, Random Forest (RF), and Logistic Regression (LR).

Dumas et al. investigated the possibility of predicting cardiac arrest by machine learning in accordance with big data development [16]. Somanchi et al. developed a cardiac arrest scoring system based on support-vector machines (SVM) using electronic medical records (EMRs) [15]. The elements of this scoring system were age, sex, race, vital signs, and laboratory data [17]. The vital signs and laboratory data were pulse oximetry, hematocrit, sodium, heart rate, systolic blood pressure (SBP), hemoglobin, potassium, alkaline phosphatase, diastolic blood pressure (DBP), glucose, magnesium, total protein, temperature, calcium, creatinine, carbon, dioxide, phosphate, platelet count, albumin, bilirubin, alanine aminotransferase, and aspartate aminotransferase [17]. Ong et al. developed a cardiac arrest model for use in critically ill patients within 72 h of presenting to the emergency department based on SVM [18]. The elements of this model were heart rate variability (HRV) with time and frequency domain, age, sex, medical history, heart rate, blood pressure, respiratory rate, Glasgow coma scale, etiology, medication history, and oxygen saturation [18]. Churpek et al. compared cardiac arrest patients to other patients in the same ward [17], and found that the maximum respiratory rate, heart rate, pulse pressure index, and minimum DBP were important predictors of cardiac arrest [19]. Churkpek et al. developed a cardiac arrest risk triage (CART) scoring system using time, temperature, blood pressure, heart rate, oxygen saturation, respiratory rate, and mental status [20]. Linu et al. developed an SVM for evaluating HRV and vital signs based on a cardiac arrest model for use within 72 h [21]. Vital signs included heart rate, temperature, SBP, DBP, pain score, Glasgow coma scale, respiratory rate, and oxygen saturation [21]. Murukesan et al. analyzed SVM and a probabilistic neural network (PNN) using HRV [22]. Kwon et al. developed a deep learning-based early warning system (DEWS) score. The DEWS score-based recurrent neural network (RNN) used four vital signs: heart rate, SBP, respiratory rate, and body temperature [15]. ElSaadyany et al. developed a wireless early prediction system of cardiac arrest through the Internet of things (IoT) using heart rate, ECG signal, body temperature, sex, age, and height [23]. The system predicted cardiac arrest using abnormal body temperature or heart rate [23]. Ueno Ryo et al. developed algorithms to predict cardiac arrest based on RF in patients [24]. They collected 8-hourly vital signs and laboratory data for two days to obtain 24-h of data [24]. Sensitivity was higher when only vital signs were used, but the use of vital signs and laboratory data gave a higher positive predictive value (PPV) [24]. Hardt et al. investigated predicted risk for clinical alerts based on deep learning using time series data [25]. Raghu et al. developed algorithms to predict clinical risk based on shallow machine learning [26]. Viton et al. developed algorithms for predictions using multivariate time series data based on deep learning in healthcare [27]. Sbrollini et al. developed a deep learning model using ECG [28]. Ibrahim et al. performed shallow and deep learning algorithms using ECG [29].

In this study, we developed and validated deep learning-based artificial intelligence algorithms for predicting adverse events including cardiopulmonary arrest, unplanned ICU transfer, and unexpected death during hospitalization in Soonchunhyang University Cheonan Hospital.

## 2. Materials

We performed a retrospective cohort study in Soonchunhyang University Cheonan Hospital, a tertiary-care teaching hospital in the Republic of Korea. The study population consisted of patients admitted to Soonchunhyang University Cheonan Hospital between January 2016 and June 2019. Table 1 shows the characteristics of our study population.

We divided the 8-h time series data from the 72-h time series data into 8-h steps. For the shallow machine learning algorithm, we split the data by shuffling the training and test data at a ratio of 9:1 and used the training data as the input for the stratified K-fold. For deep learning, we split the data by shuffling the training and test data at a ratio of 9:1. Training data was split by shuffling the training and validation data at a ratio of 9:1. Table 2 shows the input variables.

## 3. Methods

**Figure 1:**
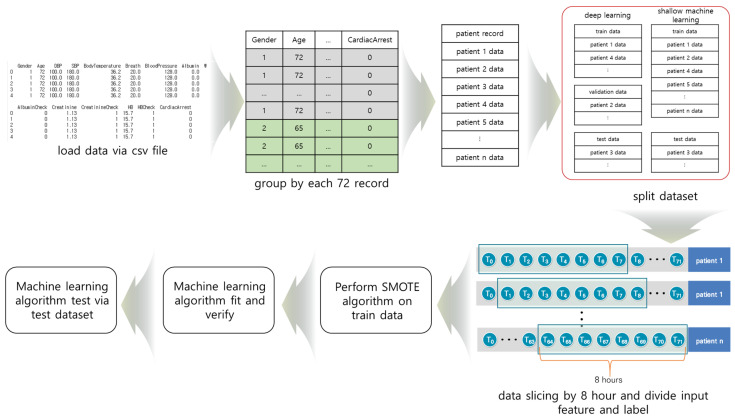
The process of cardiac arrest prediction: (1) data collection from the CSV file, (2) grouping of the data, (3) division of the data into training, verification, and test data, (4) data slicing, (5) Synthetic Minority Oversampling Technique (SMOTE) algorithm was performed on the training data, and (6) the machine learning algorithm.

We divided the data into groups of 72 h so training, verification, and test data did not mix during deep learning. The grouped data were divided into training, verification, and test data, and sliced at 8-h intervals. Figure 1 shows the cardiac arrest prediction process. We used TensorFlow, Keras, and scikit-learn for the prediction [30,31,32].

Because the Long Short-Term Memory (LSTM), Gated Recurrent Unit (GRU), and the LSTM–GRU hybrid model are deep learning models for processing sequence data in three dimensions (number of data, sequence length, and number of features), we reduced them to two dimensions (number of data, number of features) for shallow machine learning.

### 3.1. Shallow Machine Learning Model

#### 3.1.1. Decision Tree

**Figure 2:**
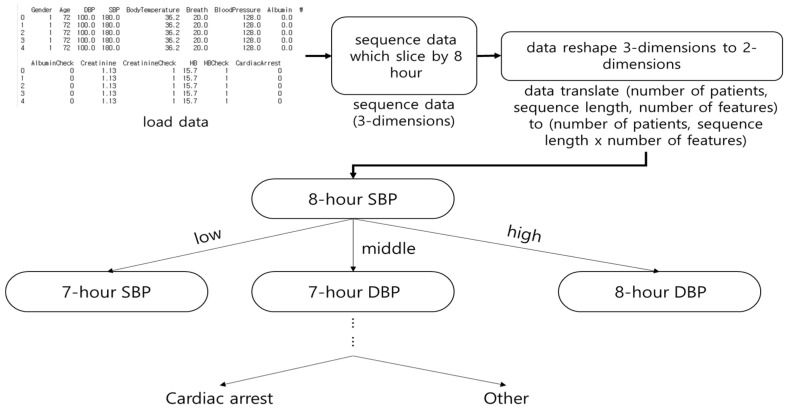
The architecture of Decision Tree (DT). The DT was classified by determining a range for all features (e.g., sex, age, pulse, and systolic blood pressure) to predict cardiac arrest.

Decision Tree (DT) was selected based on the input element. Figure 2 shows the architecture of DT. In this study, DT predicted in-hospital cardiac arrest based on 8 h of vital signs and laboratory data. DT showed the highest accuracy among the machine learning algorithms. Although time series data are expressed in three dimensions, DT has a two-dimensional input and does not consider the sequence of observations.

#### 3.1.2. Random Forest

**Figure 3:**
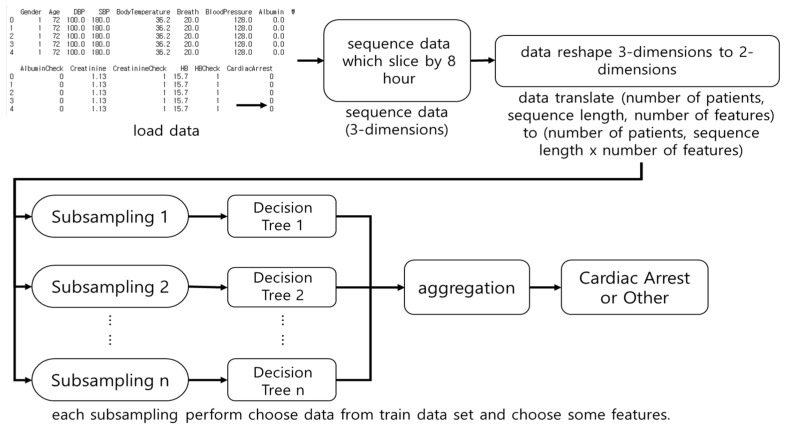
The architecture of Random Forest (RF). The RF was used as an input for multiple Decision Trees after subsampling the dataset and the results were aggregated.

RF is a DT-based ensemble model [33]. RFs sub-sample the dataset, perform DTs, and select the DT with the highest accuracy. Figure 3 shows the architecture of RF. In this study, RF performs a subsampling of 8-h of vital signs and laboratory data, and trains it on DTs. RF does not consider the sequence of observations. The number of subsamples is denoted by *n*.

#### 3.1.3. Logistic Regression

LR calculates the probability through the sigmoid function. In this study, LR calculated the weight and bias based on 8 h of vital signs and laboratory data. LR was classified by rounding off in-hospital cardiac arrest through the sigmoid function. LR input was in two dimensions so LR did not consider the sequence of observations.

### 3.2. Deep Learning

We applied the dropout technique to the deep learning model to prevent overfitting during training [34]. The dropout layer ignored some networks during training [34]. 

#### 3.2.1. Long Short-Term Memory Model

The Long Short-Term Memory (LSTM) model is an RNN model proposed by Hochreiter et al. [35]. The LSTM model solves the long-term dependency problem and considers Input gate, Forget gate, Output gate, hidden state, and long-term memory cell. 

**Figure 4:**
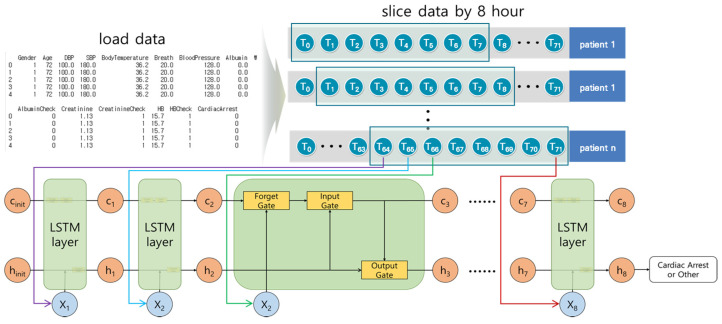
The architecture of the Long Short-Term Memory model. For each step, the input elements were trained in the LSTM model. The long-term cell (*c*) deleted unnecessary information for each step, and stored it in the long-term memory cell while learning. The hidden status was *h* (i.e., short-term memory).

Figure 4 shows the architecture of the LSTM model. In this study, the 8-h time series data were input elements used for each step. The Forget gate deleted unnecessary long-term memory by calculating previous long-term memory cells, previous outputs, and the current input elements. The Input gate calculated the current long-term memory cell. The current long-term memory was calculated using the result of the Forget gate, the current input elements, and the previous results. The Output gate calculated the short-term result using the result of the Input gate, the previous result, and the current input elements. The LSTM model is complex because of multiple variables and gates, and the execution time is slow because of the calculations in every step.

We organized the layers in the following order: LSTM layer → dropout layer → LSTM layer → dropout layer → LSTM layer → dropout layer → LSTM layer → dropout layer → dense layer. The dropout layer prevented overfitting by deactivating certain ratios during learning.

#### 3.2.2. Gated Recurrent Unit Model

The GRU model designed by Cho et al. [36] improved the processing time compared to the LSTM model. The GRU model considers the Reset and the Update gates.

**Figure 5:**
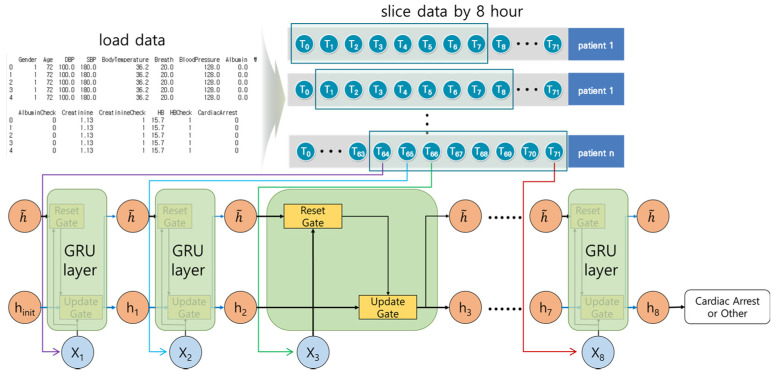
The architecture of the GRU model. For each step, the current result was calculated by considering the weights, current input elements, and previous results or temporary hidden status.

Figure 5 shows the architecture of the GRU model, which is similar to the LSTM model. It calculates the hidden state, and at each step decides to store or ignore it. The Reset gate calculates whether to consider the temporary hidden status. The Update gate calculates whether to store the current result in the temporary hidden status. The number of calculations for each step are reduced, and the structure is simpler than the LSTM model. The execution time is faster than that of the LSTM model with similar results.

We organized the layers in the following order: GRU layer → dropout layer → GRU layer → dropout layer → GRU layer → dropout layer → GRU layer → dropout layer → dense layer. For the LSTM–GRU hybrid model, we organized the layers in the following order: LSTM layer → dropout layer → LSTM layer → dropout layer → GRU layer → dropout layer → GRU layer → dropout layer → dense layer.

### 3.3. Synthetic Minority Oversampling Technique

**Figure 6:**
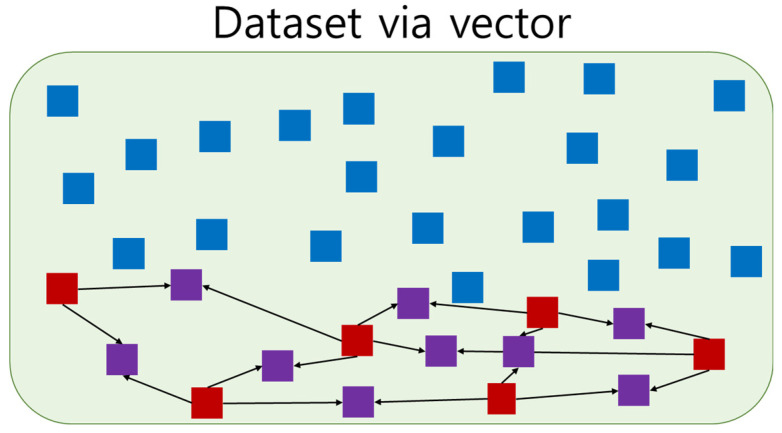
Example of Synthetic Minority Oversampling Technique. Blue is majority data, red is minority data, and purple is the generated minority data. Each generated minority data was generated from two minority data.

Cardiac arrest is less common than other cases, so our dataset was unbalanced. Under and oversampling techniques can be employed to reduce data imbalance. Undersampling decreases the majority data, and some information may be deleted [37,38]. Oversampling increases the minority data and leads to overfitting [38,39]. They should only be applied to the training dataset because they adjust datasets. Figure 6 shows the use of SMOTE based on the neighboring data; SMOTE is an oversampling technique proposed by Chawlas et al. [40].

The SMOTE algorithm was performed on each minority dataset using the K Nearest Neighbors (KNN) algorithm.

### 3.4. K-Fold Cross-Validation

K-Fold Cross-Validation is a method that cross-verifies the dataset. K-Fold Cross-Validation partitions the k data subset in the original dataset. It improves performance by verifying each partitioned dataset. The stratified K-fold Cross-Validation maintains the ratio of the majority dataset to the minority dataset. Through cross-validation, we learned not to depend on a specific partition in the learning process. We applied stratified K-fold to DT, RF, and LR, and k was set as 4, 5 and 10, respectively.

### 3.5. Material Preprocessing

For this study, we extracted raw data from the electronic health records (EHRs) of Soonchunhyang University Cheonan Hospital. Vital signs and laboratory data were measured by a medical sensor. We parsed patient information, vital signs, and laboratory data according to the measurement time for all patients from raw data. We changed the measurement interval time to an hour because measurement time intervals were different for each patient. We replaced the missing values with the last measured values. We also used 72 h data because the hospitalization period was different for each patient. Patients who were admitted or discharged outside the study period; patients under 18 years of age; patients with death or cardiac arrest within 8 h after admission were excluded. For cardiac arrest, we extracted vital signs and laboratory data for 72 h before cardiac arrest. For all other patients, we extracted vital signs and laboratory data for the first to 72 h after hospitalization.

## 4. Results

### 4.1. Performance Evaluation Method

Performance evaluation was based on the accuracy, PPV, and sensitivity. Although the evaluation methods usually use accuracy, we used PPV and sensitivity for the performance evaluation. There were four types of data prediction results: (1) true positive (TP): predicted cardiac arrest in cardiac arrest cases; (2) false positive (FP): predicted cardiac arrest in non-cardiac arrest cases (higher the FP, lower the PPV); (3) false negative (FN): did not predict cardiac arrest in cardiac arrest cases (higher the FN, lower the sensitivity); (4) true negative (TN): did not predict cardiac arrest in non-cardiac arrest cases. PPV was calculated using Equation (1)

(1) $$\mathrm{PPV}=\frac{\mathrm{TP}}{\mathrm{TP}+\mathrm{FP}}$$

Negative predictive value (NPV) was calculated using Equation (2)

(2) $$\mathrm{NPV}=\frac{\mathrm{TN}}{\mathrm{FN}+\mathrm{TN}}$$

Sensitivity was calculated using Equation (3)

(3) $$\mathrm{Sensitivity}=\frac{\mathrm{TP}}{\mathrm{TP}+\mathrm{FN}}$$

Specificity was calculated using Equation (4)

(4) $$\mathrm{Specificity}=\frac{\mathrm{TN}}{\mathrm{FP}+\mathrm{TN}}$$

In the case of classification, both PPV and sensitivity have weights, the F1 score was calculated using Equation (5), and the PPV and sensitivity were weighted at a 1:1 ratio.

(5) $$F1\;\mathrm{Score}=2\times \frac{\mathrm{PPV}\;\times \;\mathrm{Sensitivity}}{\mathrm{PPV}\;+\;\mathrm{Sensitivity}}$$

### 4.2. Performance Evaluation According to SMOTE Ratio

We used the LSTM model based on generated sequence data for each patient between 40 and 72 h for 8 h at 1-h intervals. We performed a performance evaluation using SMOTE ratio. Table 3 shows the results of the performance evaluation. The case ratio of 1:0.05 was the highest PPV.

### 4.3. Results of Shallow Machine Learning

We performed binary classification using DecisionTreeClassifier, RandomForestClassifier, and LogisticRegression provided by Scikit-learn [41,42,43]. Table 4 shows the performance evaluation based on test data.

### 4.4. Results of LSTM Model

We performed a performance evaluation based on the unit size of the LSTM model as shown in Table 5. We highlighted the highest PPV, NPV, sensitivity, specificity, and F1 scores. We decided on a unit size of 96 because it was the highest F1 score.

### 4.5. Results of GRU Model

We conducted performance evaluation using the unit size of the GRU model as shown in Table 6. We highlighted the highest PPV, NPV, sensitivity, specificity, and F1 scores. We decided on a unit size of 128 because it was the highest F1 score.

### 4.6. Results of LSTM–GRU Hybrid Model

We performed a performance evaluation based on the unit size of the LSTM–GRU model as shown in Table 7. We highlighted the highest PPV, NPV, sensitivity, specificity, and F1 scores. We decided on a unit size of 96 because it was the highest F1 score. 

### 4.7. Result of the Performance Evaluation of Shallow and Deep Learning

Based on the results of Section 4.2, we set the ratio of SMOTE to 1:0.05 in the shallow and deep learning model. We performed the shallow machine learning algorithm using a stratified K-fold algorithm with k values of 4, 5, and 10 in Section 4.3. The values of k for DT, RF, and LR were 10, 5 and 10, respectively. We performed the LSTM, GRU, and LSTM–GRU hybrid models with unit sizes 16, 32, 64, 96, and 128 in Section 4.4–4.6. The unit sizes for LSTM, GRU, and LSTM–GRU hybrid models were 96, 128, and 128, respectively. Table 8 shows the result of the performance evaluation of each algorithm. We highlighted the highest PPV, NPN, sensitivity, specificity, and F1 score.

The shallow and deep learning model had a higher PPV than the traditional EWSs. RF had the highest PPV among shallow and deep learning results. However, apart from LR, shallow and deep learning showed lower sensitivities than the traditional EWSs.

## 5. Discussion

We performed in-hospital cardiac arrest prediction based on shallow and deep learning. Sbrollini et al. [28] and Ibrahim et al. [29] developed deep learning methods for serial ECG analysis and had high performance in the detection of heart failure. However, in order to measure ECG signals, patients need to wear ECG measuring equipment. It is practically impossible for all patients to wear ECG measuring equipment for cardiac arrest. To overcome this limitation, we used vital signs and laboratory data instead of ECG for cardiac arrest prediction. Kwon et al. [15] proposed the DEWS based on vital signs. Since vital signs and laboratory data are periodically inspected to check the condition of inpatients, it is easy to obtain these data. Table 9 shows the performance of the EWS and the proposed methods in this study. Our proposed LR had the highest F1 score.

Although our proposed deep learning model had low sensitivity, it had a higher PPV than the EWS. Existing cardiac arrest prediction studies using deep learning have limitations in comparing the absolute performance of each method because the target patients are different.

The dataset in our study had two limitations. First, the data was measured at different time intervals for each patient depending on the patient’s condition. We changed the measurement interval to one hour, increasing the number of missing values that had to be replaced by the last measured value. Second, the data were collected from only Soonchunhyang University Cheonan Hospital; therefore, the study population was homogenous. In addition, the SMOTE algorithm depends on the PPV. Recently, IoT-based healthcare and hospital data management have been studied [44,45]. In the future, it is expected that improved cardiac arrest prediction models can be developed using IoT-based sensors in hospitals.

## 6. Conclusions

We proposed an in-hospital cardiac arrest prediction model based on shallow and deep learning for patients in Soonchunhyang University Cheonan Hospital. We demonstrated improved performance based on the SMOTE ratio (1:0.05). We also demonstrated improved performance based on the unit size in deep learning models (LSTM: 96; GRU: 128, and LSTM–GRU hybrid: 96). We developed an LR-based cardiac arrest prediction model that showed a better performance than the traditional EWSs. In the future, we aim to extract important features for in-hospital cardiac arrest prediction through correlation analysis to PPV and sensitivity. We plan to test our shallow and deep learning model in Soonchunhyang University Cheonan Hospital and verify the results in Soonchunhyang University Bucheon Hospital, Soonchunhyang University Seoul Hospital, and Soonchunhyang University Gumi Hospital. 

## Figures and Tables

**Table 1:** Characteristics of the study population.

Characteristics	Data
Study period	January 2016–June 2019
Total patients, n	83,543
Patients with in-hospital cardiac arrest, n	1154
Number of features, n	13
Number of data for each patient, n	72
Sequence data slice size	8
Age, years, (mean ± SD)	57.5 ± 17.0
Males, n (%)	39,428 (47.2%)
Hospital	Soonchunhyang University Cheonan Hospital

SD: standard deviation.

**Table 2:** Input variables.

Variable	Description
Age	Age at hospitalization
Sex	Man (1) or woman (2)
DBP	Diastolic blood pressure (30 ≤ DBP ≤ 300, mmHg)
SBP	Systolic blood pressure (30 ≤ SBP ≤ 300, mmHg)
Body temperature	Body temperature (30 ≤ BodyTemperature ≤ 45)
Respiratory rate	Breaths per minute (3 ≤ Breath ≤ 60)
Blood Pressure	Blood pressure (30 ≤ BloodPressure ≤ 300, mmHg)
Albumin	Albumin values (Laboratory data)
Albumin check	Presence of albumin (Present: 1, absent: 0)
Creatinine	Creatinine values (Laboratory data)
Creatinine check	Presence of creatinine (Present: 1, absent: 0)
Hb	Hemoglobin values (Laboratory data)
Hb check	Presence of HB (Present: 1, absent: 0)

SBP: systolic blood pressure; DBP: diastolic blood pressure; Hb: hemoglobin.

**Table 3:** Performance evaluation for the SMOTE ratio. The highest positive predictive value was observed at a ratio of 1:0.05 and highest negative predictive value was observed at a ratio of 1:0.07. The highest sensitivity was observed at a ratio of 1:0.07 and highest specificity was observed at a ratio of 1:0.05. The highest F1 score was observed at a ratio of 1:0.07.

Ratio	PPV	NPV	Sensitivity	Specificity	F1 Score
1:1	20.78%	99.12%	37.46%	98.01%	26.73%
1:0.08	38.14%	99.10%	35.17%	99.20%	36.59%
1:0.07	41.47%	99.20%	42.43%	99.16%	41.95%
1:0.06	41.49%	99.14%	38.57%	99.24%	39.98%
1:0.055	39.83%	99.14%	38.16%	99.20%	38.98%
1:0.05	43.28%	99.11%	35.86%	99.34%	39.22%
1:0.045	36.13%	99.08%	33.74%	99.17%	34.89%
1:0.025	36.82%	99.06%	32.80%	99.21%	34.69%

PPV: positive predictive value; NPV: negative predictive value.

**Table 4:** Performance evaluation of shallow machine learning performed by changing the value of k in the stratified K-fold. The highest PPVs in DT, RF, and LR were 10, 5, and 10, respectively.

Algorithm	K	PPV	NPV	Sensitivity	Specificity	F1 Score
DT	4	43.99%	98.97%	25.80%	99.54%	32.52%
	5	45.02%	98.98%	26.67%	99.55%	33.50%
	10	46.80%	99.01%	28.99%	99.54%	35.80%
RF	4	97.20%	98.94%	23.48%	99.99%	37.82%
	5	98.22%	98.95%	24.25%	100.00%	38.94%
	10	96.44%	98.98%	26.18%	99.99%	41.19%
LR	4	5.12%	99.57%	75.07%	80.60%	9.59%
	5	5.12%	99.57%	74.98%	80.60%	9.58%
	10	5.14%	99.57%	76.33%	80.35%	9.64%

DT: Decision Tree; RF: Random Forest; LR: Logistic Regression; PPV: positive predictive value; NPV: negative predictive value.

**Table 5:** Performance evaluation of LSTM model. We evaluated the performance according to LSTM layer unit size. The highest PPV was 96 and highest NPV was 32. The highest sensitivity was 64 and highest specificity was 96. The highest F1 score was 96.

Unit Size	PPV	NPV	Sensitivity	Specificity	F1 Score
16	27.77%	99.05%	31.98%	98.84%	29.73%
32	33.80%	99.08%	32.56%	99.11%	33.17%
64	32.71%	99.06%	34.01%	99.02%	33.35%
96	38.37%	99.06%	32.66%	99.27%	35.28%
128	35.45%	99.06%	32.46%	99.18%	33.91%

PPV: positive predictive value; NPV: negative predictive value.

**Table 6:** Performance evaluation of the GRU model according to the GRU layer unit size. The highest PPV was 128 and highest NPV was 32. The highest sensitivity was 32 and highest specificity was 128. The highest F1 score was 128.

Unit Size	PPV	NPV	Sensitivity	Specificity	F1 Score
16	26.28%	99.07%	33.62%	98.68%	29.50%
32	28.75%	99.19%	42.61%	98.53%	34.33%
64	32.05%	99.11%	36.33%	98.93%	34.06%
96	32.05%	99.11%	36.33%	98.93%	32.50%
128	34.59%	99.09%	34.59%	99.09%	34.69%

PPV: positive predictive value; NPV: negative predictive value; GRU: gated recurrent unit.

**Table 7:** Performance evaluation of the LSTM–GRU model according to the GRU and LSTM layer unit sizes. The highest PPV was 128 and highest NPV was 96. The highest sensitivity was 96 and highest specificity was 16. The highest F1 score was 96.

Unit Size	PPV	NPV	Sensitivity	Specificity	F1 Score
16	31.79%	98.62%	22.51%	99.33%	26.36%
32	23.34%	99.06%	33.33%	98.47%	27.46%
64	27.39%	99.06%	32.66%	98.79%	29.79%
96	30.53%	99.14%	38.65%	98.77%	34.12%
128	35.30%	99.06%	32.37%	99.17%	33.77%

PPV: positive predictive value; NPV: negative predictive value.

**Table 8:** Performance evaluation of shallow and deep learning. The highest PPV was DT, highest NPV was LR, highest sensitivity was LR, highest specificity was RF, and highest F1 score was RF.

Algorithm	PPV	NPV	Sensitivity	Specificity	F1 Score
DT	46.80%	99.01%	28.99%	99.54%	35.80%
RF	98.22%	98.95%	24.25%	100.00%	38.94%
LR	5.14%	99.57%	76.33%	80.35%	9.64%
LSTM model	38.37%	99.06%	32.66%	99.27%	35.28%
GRU model	34.59%	99.09%	34.59%	99.09%	34.69%
LSTM–GRU hybrid model	30.53%	99.14%	38.65%	98.77%	34.12%

DT: Decision Tree; RF: Random Forest; LR: Logistic Regression; PPV: positive predictive value; NPV: negative predictive value.

**Table 9:** Results of EWS and our methods. Our proposed LR had higher PPV and sensitivity than traditional EWS.

Algorithm		PPV	NPV	Sensitivity	Specificity	F1 Score
Traditional EWS [15]	SPTTS	0.4%	99.9%	60.7%	77.0%	0.8%
	MEWS ≥ 3	0.5%	99.9%	63.0%	79.9%	1.0%
	MEWS ≥ 4	0.6%	99.9%	49.3%	86.8%	1.2%
	MEWS ≥ 5	0.6%	99.9%	37.3%	90.6%	1.3%
Joon-myoung Kwon et al. [15]	RF	0.4%	99.9%	75.3%	69.9%	0.8%
	LR	0.2%	99.9%	76.3%	34.6%	0.4%
	DEWS ≥ 2.9	0.5%	99.9%	75.7%	76.5%	1.0%
	DEWS ≥ 3	0.5%	99.9%	75.3%	77.0%	1.0%
	DEWS ≥ 7.1	0.8%	99.9%	63.0%	87.0%	1.5%
	DEWS ≥ 8.0	0.8%	99.9%	60.7%	88.3%	1.6%
	DEWS ≥ 18.2	1.4%	99.9%	49.3%	94.6%	2.8%
	DEWS ≥ 52.8	3.7%	99.9%	37.3%	98.4%	7.1%
Ueno Ryo et al. [24]	RF (vital signs, medical patients)	0.47%	99.7%	80.30%	78.30%	0.9%
	RF (vital signs and lab data, medical patients)	0.52%	99.7%	79.60%	80.90%	1.0%
Ibrahim Lujain et al. [29]	CNN model	-	-	88.1%	93.2%	89.9%
	RNN model	-	-	78.0%	87.8%	82.2%%
	XGBoost	-	-	93.5%	99.4%	97.1%
Our methods	DT	46.80%	99.01%	28.99%	99.54%	35.80%
	RF	98.22%	98.95%	24.25%	100.00%	38.94%
	LR	5.14%	99.57%	76.33%	80.35%	9.64%
	LSTM model	38.37%	99.06%	32.66%	99.27%	35.28%
	GRU model	34.59%	99.09%	34.59%	99.09%	34.69%
	LSTM–GRU hybrid model	30.53%	99.14%	38.65%	98.77%	34.12%

MEWS: modified early warning score; DEWS: deep learning-based early warning system; DT: Decision Tree; RF: Random Forest; LR: Logistic Regression; PPV: positive predictive value; NPV: negative predictive value.

## Data Availability

Data sharing not applicable.
